# Beyond the Valve: Redefining Post-Transcatheter Aortic Valve Replacement Care With Sodium-Glucose Cotransporter-2 Inhibition

**DOI:** 10.1016/j.shj.2026.100821

**Published:** 2026-02-16

**Authors:** Claire Graves, Michael I. Brener

**Affiliations:** a Department of Medicine, Columbia University Irving Medical Center, New York, New York, USA; b Cardiovascular Research Foundation, New York, New York, USA

Aortic stenosis (AS) is a mechanical problem in need of a mechanical solution (i.e., aortic valve replacement); this has been the conventional wisdom underlying management of AS for decades. Accordingly, transcatheter aortic valve replacement (TAVR) is often viewed as the terminal therapeutic goal for patients with severe AS. Once the valve is replaced, medical therapy and management of residual comorbidities are largely overlooked. However, patients with severe AS post TAVR continue to be at high risk for persistent heart failure (HF), progressive renal dysfunction, and ongoing cardiometabolic derangements. The elevated risk can be attributed to advanced (and frequently irreversible) myocardial fibrosis, left ventricular hypertrophy, diastolic dysfunction, and adverse cardiac remodeling.^1^^,^^2^ These mechanisms provide a clear need for extending disease-modifying medical therapy into the post-TAVR period.

Sodium-glucose cotransporter-2 inhibitors (SGLT2i) have become an integral therapy for several different cardiovascular conditions, including HF with preserved and reduced ejection fraction. Although most patients with severe AS being considered for intervention were excluded from the large randomized trials evaluating SGLT2i (i.e., DAPA-HF,^3^ EMPEROR-Reduced,^4^ and EMPEROR-Preserved^5^), the DAPA-TAVI trial showed that dapagliflozin significantly reduced the risk of all-cause mortality or worsening HF compared to standard care among individuals with severe AS undergoing TAVR. This was primarily driven by fewer HF events.^6^

However, the DAPA-TAVI trial investigated dapagliflozin in a more restricted trial population, typical of randomized trials. Eligible participants had to have HF symptoms attributed to their AS with comorbid renal insufficiency, diabetes mellitus, or reduced left ventricular ejection fraction (LVEF). Whether SGLT2i have similar efficacy in the broader, more heterogenous population of individuals with severe AS being considered for TAVR remains unknown.

In this context, Zahid et al leveraged a large, real-world database to examine the association between SGLT2i use and clinically relevant outcomes after TAVR.^7^ Using the TriNetX Research Network, the authors identified 61,763 adults who underwent TAVR between 2013 and 2024. Of those patients, only 3529 (5.7%) were prescribed SGLT2i before or within 1 month of TAVR. Following propensity score matching, 3362 well-balanced patient pairs were analyzed. The primary outcome was all-cause mortality assessed at 6 months, 1 year, and 3 years post TAVR. The secondary outcomes were hospitalization, HF exacerbation, acute kidney injury, genitourinary infections, adverse events, and renal replacement therapy assessed at the same time points.

Several features of this analysis enhance its relevance. First, the size and extended study period of the cohort provide insight into the evolution of routine clinical care over a decade. Second, the extended follow-up of 3 years offers key insights into the durability of potential benefits from SGLT2i by addressing a knowledge gap in a population with significant late morbidity and mortality. Lastly, with the study’s liberal inclusion criteria, the authors captured patients that may otherwise be excluded from randomized trials. This allows the analysis to expand the evidence basis supporting SGLT2i use after TAVR.

SGLT2i use was associated with significantly lower all-cause mortality at all time points with a 3-year mortality of 13.2% compared to 19.4% among matched controls (*p* < 0.001). Rates of hospitalization and HF exacerbations were also significantly reduced in the SGLT2i group at all time points. Over the 3-year study period, the rate of HF exacerbations was 35%, significantly higher than reported in previous randomized trials. As acknowledged by the authors, this may be a result of differences in event identification given that the database relies on administrative coding rather than adjudicated endpoints.

Multiple significant differences in both primary and secondary outcomes were identified in subgroup analyses, as well. For patients with stage IV chronic kidney disease, SGLT2i use was associated with significantly lower mortality 1 and 3 years post TAVR. Patients with type 2 diabetes experienced reductions in mortality and hospitalizations at all time points. Similarly, in patients with LVEF <45% and LVEF ≥45%, SGLT2i use was associated with reduced mortality, and in patients with LVEF ≥45%, SGLT2i use was also associated with reduced hospitalization and HF exacerbations.

There are several important takeaways from this study. First, patients with AS frequently suffer from persistent HF even after TAVR, as evidenced by the high rate of HF exacerbations in this real-world registry. Second, SGLT2i remain markedly underused in this vulnerable population, with less than 6% of patients receiving therapy; this underscores a major opportunity to improve post-TAVR care. Given the magnitude and consistency of benefit observed across multiple clinically relevant endpoints, these findings argue for a paradigm shift in post-TAVR management—one that integrates structured medical optimization alongside procedural success. Finally, although this large observational analysis complements randomized trial data and enhances generalizability, important limitations inherent to these large administrative datasets must be noted. The use of these datasets may result in possible confounding, misclassification, and incomplete capture of clinical detail necessary for understanding implementation of medical therapies in new populations. If these limitations are acknowledged, then together, these findings strengthen the rationale for prospective trials to define the role, timing, and durability of SGLT2i as a foundational component of post-TAVR management.

## Funding

Michael Brener was supported by 1K23HL177311-01A1 and has received grant funding from Abbott Vascular as well as consulting honoraria from Edwards Lifesciences, CroiValve, Approxima, and Ventricord.

## Disclosure Statement

The authors report no conflict of interest.
